# Expression of the potential therapeutic target claudin-18.2 is frequently decreased in gastric cancer: results from a large Caucasian cohort study

**DOI:** 10.1007/s00428-019-02624-7

**Published:** 2019-07-22

**Authors:** Matthias Dottermusch, Sandra Krüger, Hans-Michael Behrens, Christine Halske, Christoph Röcken

**Affiliations:** Department of Pathology, Christian-Albrechts-University, Arnold-Heller-Str. 3, Haus 14, 24105 Kiel, Germany

**Keywords:** Gastric cancer, Stomach neoplasms, Tight junctions, Claudin-18.2, EBV, Prognosis

## Abstract

Gastric cancer (GC) is frequently diagnosed and treated in advanced tumour stages with poor prognosis. Recent studies have identified isoform 2 of the tight junction protein claudin-18 (CLDN18.2) as a promising target in GC therapy. In this study, we aimed to outline the expression of CLDN18.2 and its correlation with clinico-pathological patient characteristics in a large and well-characterized cohort of GC patients. The expression of CLDN18.2 was studied in 481 GCs by immunohistochemistry on whole tissue sections. Immunostained GCs were evaluated using the histoscore (H-score) and subsequently divided into two groups: tumours showing any or no expression. CLDN18.2 expression was investigated for correlation with various clinico-pathological patient characteristics, including survival. CLDN18.2 expression was found in 203 GCs (42.2%). Of these tumours, 71 (14.8%) showed solely weak immunostaining. CLDN18.2 expression correlated with mucin phenotype, EBV status, the integrin αvβ5, the EpCAM extracellular domain EpEX, and lysozyme. We found no correlation with survival, Laurén phenotype, or any other clinico-pathological patient characteristic. In conclusion, we demonstrate frequently decreased expression of CLDN18.2 in a GC cohort of appropriate size. Correlating CLDN18.2 expression with clinico-pathological patient characteristics reveals new linkages to αvβ5, EpEX, and lysozyme, which may pave the way for further investigations regarding the role of tight junction proteins in GC progression. Though CLDN18.2 continues to pose an attractive target candidate, we conclude that a rather low overall expression rate challenges its significance in advanced GC therapy and indicates the need for further investigations across different populations.

**Electronic supplementary material:**

The online version of this article (10.1007/s00428-019-02624-7) contains supplementary material, which is available to authorized users.

## Introduction

Despite the extensive geographical variability of its incidence, gastric cancer (GC) is acknowledged as one of the most common malignancies worldwide. Unfortunately, GC is frequently discovered in advanced stages, aggravating its poor prognosis [1, 2]. Furthermore, the results of systemic chemotherapy are often limited due to therapeutic aggressiveness and poor performance status of patients [3]. This calls for the development of new targeted agents to extend treatment possibilities while reducing substantial side effects of systemic therapy.

Claudin-18 (CLDN18) is a member of the claudin family and a component of tight junctions, regulating paracellular barrier functions. The expression of the isoform 2 of CLDN18 (CLDN18.2) has been shown to be restricted to differentiated epithelial cells of the gastric mucosa and also primary gastric malignancies, hence emphasizing its potential as a candidate in targeted therapy. Of note, ectopic expression is frequently found in other malignant entities such as pancreatic, oesophageal, ovarian, and lung tumours [4].

The phase II-FAST study (NCT01630083) investigated CLDN18.2 tumour expression and therapy with the chimeric monoclonal anti-CLDN18.2 antibody IMAB362 in combination with first-line chemotherapy in patients with advanced cancer of the stomach and gastro-oesophageal junction. It was shown that adding IMAB362 to chemotherapy improved overall- (OS) and progression-free survival (PFS) as well as objective response rate (ORR) in patients and therefore, CLDN18.2 was identified as a promising novel treatment target [5].

Yet, little is known about the clinical and pathological characteristics of CLDN18.2 expression in the wide field of heterogeneous GCs and across cohorts of different ethnic groups. Therefore, we investigated the expression of CLDN18.2 in a large and extensively characterized Caucasian cohort of therapy-naive GCs.

## Materials and methods

### Study population

From the archive of the Institute of Pathology, University Hospital Kiel, we sought all patients who had undergone either total or partial gastrectomy for adenocarcinoma of the stomach or oesophago-gastric junction between 1997 and 2009. We included patients with histologically confirmed adenocarcinoma of the stomach or gastro-oesophageal junction. Patients were excluded if histology of a tumour type other than adenocarcinoma was identified or if patients had undergone a perioperative or neoadjuvant chemo- or radiotherapy. Each resected specimen had undergone gross sectioning and histological examination by trained and board-certified surgical pathologists. Dates of patients’ deaths were obtained from the Epidemiological Cancer Registry of the state of Schleswig-Holstein, Germany. Follow-up data of those patients who were still alive were retrieved from hospital records and general practitioners.

### Histology

Tissue specimens were fixed in formalin and embedded in paraffin. Deparaffinized sections were stained with haematoxylin and eosin. Histological re-examination of primary tissue sections was carried out for all cases to assure if inclusion criteria were met.

### Immunohistochemistry

Immunohistochemical CLDN18.2 stainings of GCs were carried out with a Bondmax automated slide staining system (Leica Biosystems, Wetzlar, Germany), using the Polymer Refine Detection Kit (Leica Biosystems) and the anti-CLDN18.2 antibody (clone EPR19202**,** Abcam, Cambridge, UK) in a 1:200 dilution. Pretreatment was done with ER-2 (Leica Biosystems) for 20 min.

### Scoring of CLDN18.2 staining

Scoring of each tumour was assessed by determining a histoscore (H-score), following a semi-quantitative approach combining both immunostaining intensities (subsequently referred to as IHC-scores) and percentages of positive cells of the tumour. The IHC-score was based on tumour cells showing either strong (3+), intermediate (2+), weak (1+), or no (0) membranous staining of CLDN18.2. IHC-score 3+ was given if strong staining was circumferentially present in tumour cells. Partially present strong staining or circumferential light staining was assessed with the IHC-score 2+. If faint staining was partially present, score 1+ was given. Tumour cells without detectable membranous staining were scored with 0. The percentage of positive tumour cells (approximated to the nearest 10) showing the defined staining intensities (3+, 2+, 1+, 0) was gauged with respect to all tumour cells visible on each tissue specimen and always added up to a total of 100% tumour cells. Finally, a H-score was calculated according to the formula: H-score = [0 × percentage of immunonegative tumour cells] + [1 × percentage of weakly stained tumour cells] + [2 × percentage of intermediately stained tumour cells] + [3 × percentage of strongly stained tumour cells]. The maximum possible H-score was 300, if all cells of a given tumour sample showed a strong staining: [0 × 0%] + [1 × 0%] + [2 × 0%] + [3 × 100%] = 300. The multipliers within the formula yield an improved stratification of the H-scores: tumour samples with a predominantly high staining intensity and such samples with a predominantly low staining intensity are more distinctively separated.

### Assessment of heterogeneous expression

Upon reviewing both the IHC- and the H-scores, intratumoural heterogeneity becomes readily evident. Since there is no general guideline to assess heterogeneity in GC, we considered tumours to show a strong heterogeneity phenotype, when both 3+ and 0 IHC-scores were detectable and made up at least 50% of the tumour tissue combined. Heterogeneous tumours were additionally assessed for immunostaining patterns. Some GCs showed an apparent decrease in immunostaining intensity towards the depths of the tumour, which we referred to as a “downward gradient”. Heterogeneity pattern of tumours with diffusely distributed tumour cells with low or no staining was considered “scattered”. Tumours with a “patchy” pattern predominantly displayed large randomly distributed and well-circumscribed areas of aggregated tumour cells with low or no staining.

### Assessment of further clinico-pathological characteristics

The assessment of mucin expression was carried out with monoclonal antibodies directed against mucin 2 (clone Ccp58, 1:100; Novocastra, Leica Microsystems GmbH, Wetzlar, Germany), mucin 5 (clone 45 M1, 1:100; Thermo Scientific, Schwerte, Germany), mucin 6 (clone CLH5, 1:100), and CD10 (clone 56C6, 1:10; both Novocastra) [6]. Tumours were subsequently classified according to mucin phenotype [7]. pTNM stage of all study patients was determined according to the 8th edition of the UICC guidelines [8]. Infection with *H. pylori* was evaluated histologically using the modified Giemsa staining and PCR. *H. pylori*–specific DNA sequences were detected by a PCR-based assay targeting the 16S rRNA gene of *H. pylori* [6]. Epstein-Barr virus–encoded RNA was detected using the EBER probe (Novocastra) and the BondMax detection system according to the manufacturer’s instructions (Leica Microsystems GmbH) [9]. MSI status was assessed by immunohistochemistry using antibodies directed against MLH1, PMS2, MSH2, and MSH6. For each case with reduced or absent nuclear staining, subsequent molecular comparison of the allelic profiles of the mononucleotide repeat markers BAT-25, BAT-26, NR-21, NR-24, and NR-27 in tumour and corresponding normal tissue was carried out [10]. Tumours were classified according to the Laurén classification [11]. Furthermore, assessment of EpEX, EpICD, E-cadherin, αvβ3, αvβ5, and lysozyme, as well as HER2 and MET status expression, was performed as previously described [6, 9, 10, 12–15].

### Statistical analysis

SPSS version 25.0.0.2 (IBM Corp., Armonk, NY, USA) was used for statistical analyses. To test for correlation between non-ordinal variables, we used Fisher’s exact test. When testing for correlation between variables of ordinal scale, we used Kendall’s tau test. We assumed a significance level of 0.05. To compensate false discovery rate within the correlations, we applied the Simes (Benjamini-Hochberg) procedure (multiple testing correction). We also included all variables with *p* < 0.1 into a binary logistic regression (multivariate analysis) to test for independence. All *p* values having lost significance were marked accordingly. Median survival with 95% confidence intervals was determined by the Kaplan-Meier method. Differences between median survivals were tested with the log-rank test. A multivariate survival analysis (Cox regression) was performed. All *p* values are given uncorrected.

## Results

Table 1 summarizes the clinico-pathological patient characteristics of the GC cohort. Four hundred eighty-one patients fulfilled all the study criteria.

**Table 1:** CLDN18.2 expression and correlation with clinico-pathological patient characteristics

Characteristic	Variable	Total valid	CLDN18.2 expression		*p* value
		[*N* (%)]	Positive [*N* (%)]	Negative [*N* (%)]	
Age	< 68	234 (49.4)	108 (46.2)	126 (53.8)	0.094
	≥ 68	240 (50.6)	92 (38.3)	148 (61.7)	
Gender	Female	179 (37.2)	72 (40.2)	107 (59.8)	0.506
	Male	302 (62.8)	131 (43.4)	171 (56.6)	
Localization	Proximal	145 (31.0)	60 (41.4)	85 (58.6)	0.840
	Distal	323 (69.0)	137 (42.4)	186 (57.6)	
Laurén phenotype	Intestinal	252 (52.5)	114 (45.2)	138 (54.8)	0.167
	Diffuse	145 (30.2)	55 (37.9)	90 (62.1)	
	Mixed	31 (6.5)	16 (51.6)	15 (48.4)	
	Unclassified	52 (10.8)	17 (32.7)	35 (67.3)	
Mucin type	Intestinal	122 (29.0)	29 (23.8)	93 (76.2)	< 0.001*
	Gastric	64 (15.2)	32 (50.0)	32 (50.0)	
	Mixed	162 (38.5)	78 (48.1)	84 (51.9)	
	Unclassified	73 (17.3)	41 (56.2)	32 (43.8)	
T category	T1	58 (12.1)	25 (43.1)	33 (56.9)	0.703
	T2	53 (11.0)	21 (39.6)	32 (60.4)	
	T3	195 (40.6)	81 (41.5)	114 (58.5)	
	T4	174 (36.3)	76 (43.7)	98 (56.3)	
N category	N0	136 (28.5)	60 (44.1)	76 (55.9)	0.758
	N1	67 (14.0)	27 (40.3)	40 (59.7)	
	N2	84 (17.6)	29 (34.5)	55 (65.5)	
	N3	190 (39.8)	86 (45.3)	104 (54.7)	
M category	0	387 (80.5)	159 (41.1)	228 (58.9)	0.352
	1	94 (19.5)	44 (46.8)	50 (53.2)	
UICC stage	I	78 (16.4)	33 (42.3)	45 (57.7)	0.716
	II	107 (22.4)	46 (43)	61 (57)	
	III	198 (41.5)	79 (39.9)	119 (60.1)	
	IV	94 (19.7)	44 (46.8)	50 (53.2)	
L category	L0	214 (48.1)	88 (41.1)	126 (58.9)	0.773
	L1	231 (51.9)	99 (42.9)	132 (57.1)	
V category	V0	394 (88.7)	160 (40.6)	234 (59.4)	0.225
	V1	50 (11.3)	25 (50.0)	25 (50.0)	
Grading	G1/G2	116 (24.3)	49 (42.2)	67 (57.8)	1.000
	G3/G4	361 (75.7)	153 (42.4)	208 (57.6)	
R status	R0	402 (87.2)	160 (39.8)	242 (60.2)	0.023^1,2^
	R1 & 2	59 (12.8)	33 (55.9)	26 (44.1)	
*H. pylori* status	Negative	341 (84.8)	143 (41.9)	198 (58.1)	1.000
	Positive	61 (15.2)	26 (42.6)	35 (57.4)	
EBV status	Negative	445 (95.5)	179 (40.2)	266 (59.8)	< 0.001*
	Positive	21 (4.5)	17 (81.0)	4 (19.0)	
MSI status	MSS	429 (92.3)	179 (41.7)	250 (58.3)	1.000
	MSI	36 (7.7)	15 (41.7)	21 (58.3)	
HER2 status	Negative	400 (92.2)	173 (43.3)	227 (56.8)	0.029^1^
	Positive	34 (7.8)	8 (23.5)	26 (76.5)	
MET status	Negative	434 (92.9)	180 (41.5)	254 (58.5)	0.277
	Positive	33 (7.1)	17 (51.5)	16 (48.5)	
EpEX	Negative	315 (70.6)	149 (47.3)	166 (52.7)	< 0.001*
	Positive	131 (29.4)	38 (29.0)	93 (71.0)	
EpICD	Negative	142 (31.6)	64 (45.1)	78 (54.9)	0.472
	Positive	307 (68.4)	126 (41.0)	181 (59.0)	
E-cadherin	Negative	324 (73.1)	132 (40.7)	192 (59.3)	0.387
	Positive	119 (26.9)	54 (45.4)	65 (54.6)	
αvβ3 integrin	Negative	336 (74.2)	142 (42.3)	194 (57.7)	0.588
	Positive	117 (25.8)	46 (39.3)	71 (60.7)	
αvβ5 integrin	Negative	209 (46.3)	72 (34.4)	137 (65.6)	0.007*
	Positive	242 (53.7)	114 (47.1)	128 (52.9)	
Lysozyme	Negative	217 (50.5)	66 (30.4)	151 (69.6)	< 0.001*
	Positive	213 (49.5)	115 (54.0)	98 (46.0)	

*EBV*, Epstein-Barr virus; *MSI*, microsatellite instability

*p* values obtained via Fisher’s exact test or Kendall’s tau test.; *Significant after multiple testing correction

^1^Not significant after multiple testing correction. ^2^Not significant after multivariate analysis

### CLDN18.2 expression rate

**Fig. 1:**
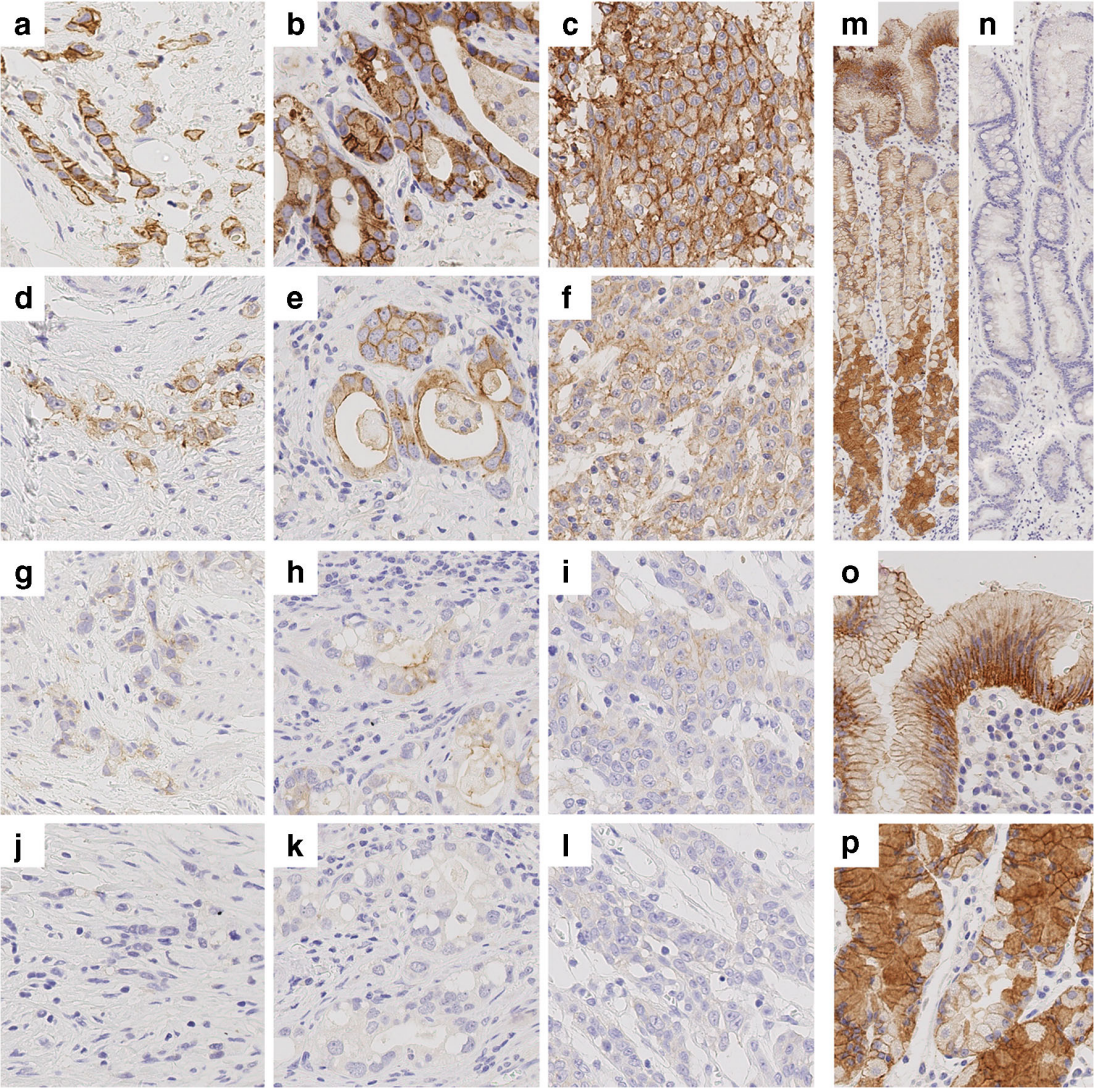
CLDN18.2 expression in neoplastic and non-neoplastic gastric cells. **a**–**l** Representative images of CLDN18.2 expression in gastric cancer cells with different growth patterns: diffuse (**a**, **d**, **g**, **j**), intestinal (**b**, **e**, **h**, **k**), and unclassified (**c**, **f**, **i**, **l**). Immunostaining of tumour cells was scored as 3+/ strong (**a**–**c**), 2+/ intermediate (**d**–**f**), 1+/ weak (**g**–**i**), or 0/ none (**j**–**l**). **m**–**p** Strong CLDN18.2 expression was seen in foveolar und glandular epithelium of the normal gastric mucosa with sparse staining in the neck region (**m**). Foveolar epithelium (**o**) displayed a strong membranous staining predominantly in the basolateral region. Glandular epithelium (**p**) further displayed cytoplasmic staining with granular morphology. Intestinal metaplasia (**n**) was regularly devoid of CLDN18.2 expression. Original magnification 400-fold (**a**–**l**, **o**, **p**) and 100-fold (**m**, **n**)

**Fig. 2:**
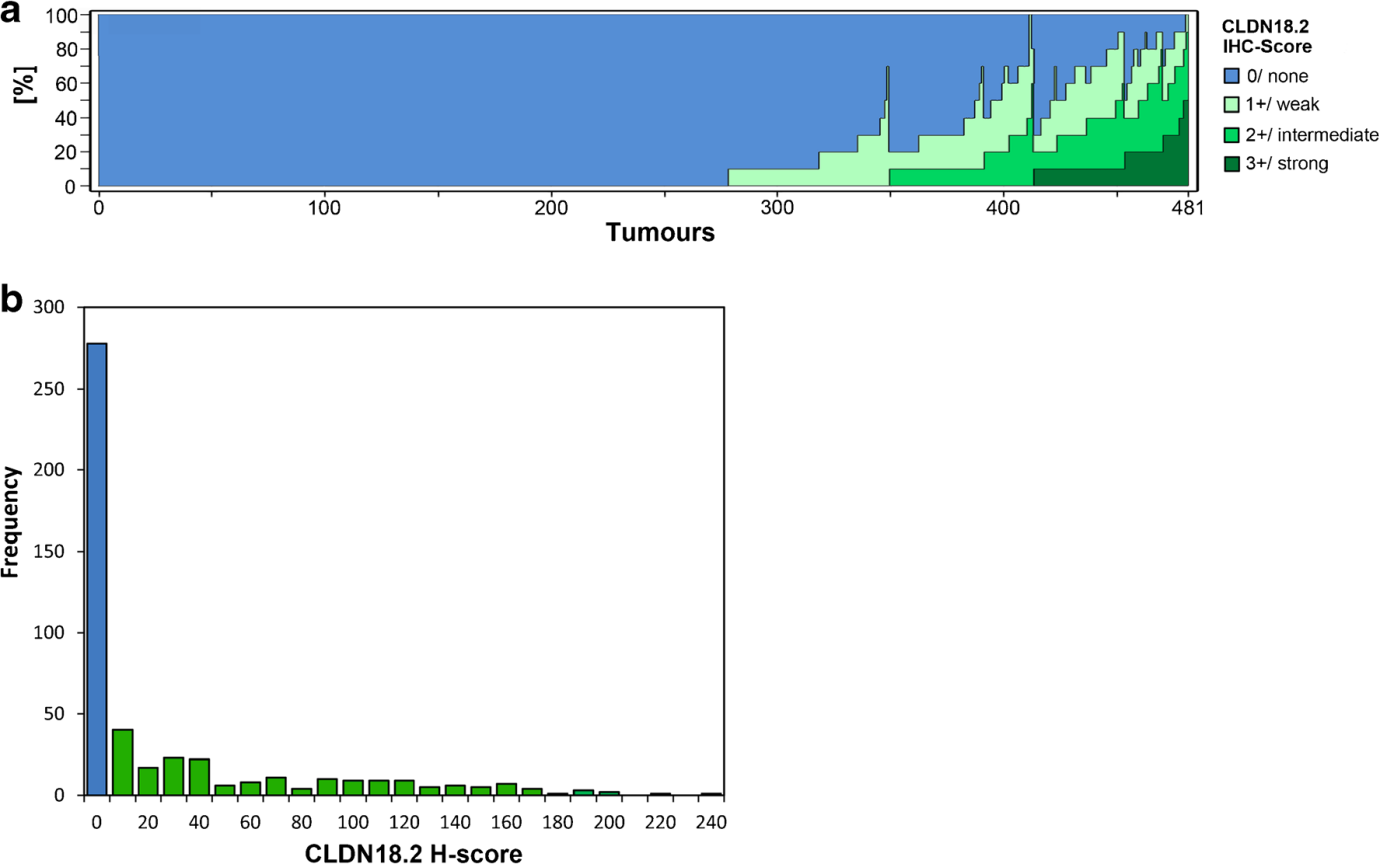
CLDN18.2 overall expression intensity. **a** 278 of 481 gastric carcinomas (57.8%) were completely devoid of any CLDN18.2 expression. In the positive cases, the maximum staining intensity reached was either weak (71 cases, 14.8%), intermediate (64 cases, 13.3%), or strong (68 cases, 14.1%). **b** The distribution of H-scores in the cohort is shown. Minimum H-Score is 0. Maximum H-score reached is 240. Median H-score of positive tumours is 40

The expression was studied using whole tissue sections. CLDN18.2 was observed in non-neoplastic gastric mucosa and tumour cells. GC was considered positive, if membranous staining was visible in tumour cells (Fig. 1a–l). Normal, non-neoplastic gastric mucosa consistently displayed strong membranous and cytoplasmic staining (Fig. 1m, o, p). In conformity with similar findings [16], we observed loss of CLDN18.2 expression in intestinal metaplasia, whenever it was present (Fig. 1n). The overall expression rate of CLDN18.2 in tumour cells was rather low: 278 (57.8%) GCs were completely devoid of any CLDN18.2 expression. Of the positive GCs, 71 (14.8%) were scored no higher than IHC 1+, while 64 (13.3%) were scored up to but no higher than IHC 2+, and 68 cases (14.1%) were scored up to IHC 3+. Maximum of CLDN18.2-IHC 3+ found was in 50% of tumour cells, observable in two cases. Immunonegative tumour cells (CLDN18.2-IHC 0) were found in 479 (99.6%) cases. Distribution of IHC-scores in the cohort is displayed in Fig. 2a. Figure 2b summarizes the frequency and distribution of CLDN18.2-H-scores.

In order to find correlations of CLDN18.2 expression with clinico-pathological patient characteristics, the cohort was dichotomized by the median score, which corresponded to 0, due to the rather low overall staining intensity observed. Thus, the cohort was split into GCs with and without visible CLDN18.2 expression.

### GC subtypes express CLDN18.2 differentially

CLDN18.2 expression correlated with mucin phenotype (*p* < 0.001), lysozyme expression (LYZ; *p* < 0.001), and EBV status (*p* < 0.001). Mucin phenotypes had been assessed in 421 cases. In our cohort, GCs showed either intestinal (122 cases, 29%), gastric (64 cases, 15.2%), mixed (162 cases, 38.5%), or unclassified (73 cases, 17.3%) mucin differentiation. One hundred eighty of 421 GCs were CLDN18.2-positive; these showed the following distribution of mucin phenotypes: intestinal 29 cases (16.1% of CLDN18.2-positive tumours), gastric 32 cases (17.8%), mixed 78 cases (43.3%), and unclassified 41 cases (22.8%). Most notably, CLDN18.2 expression is predominantly deviant in GCs of the intestinal phenotype (93 of 122 intestinal GCs (76.2%) were CLDN18.2-negative).

Of 430 GCs, 213 (49.5%) were categorized as LYZ-positive. CLDN18.2 and LYZ expression significantly correlated (*p* < 0.001), with 181 CLDN18.2-positive GCs showing LYZ expression in 115 cases (63.5%). However, no correlation was found between CLDN18.2 and the Laurén phenotype.

In our cohort, 21 of 466 GCs were EBV-positive. CLDN18.2 expression correlated with positive EBV status on a highly significant level (*p* < 0.001). Furthermore, EBV-positive CLDN18.2-positive GCs tended to show strong CLDN18.2 expression more frequently. In numbers, only 4 of 21 EBV-positive GCs (19%) were devoid of any CLDN18.2 expression, while 11 of 21 EBV-positive GCs (52.4%) were scored up to IHC 3+. We found no correlation between CLDN18.2 and MSI status.

### CLDN18.2 and further tumour or patient characteristics

We found no correlation with HER2 and MET status (Table 1). No further clinico-pathological patient characteristic, i.e. gender, age, localization, TNM, or tumour stage, correlated with CLDN18.2 expression.

### CLDN18.2 correlates with other cell junction proteins

The expression of the integrin αvβ5 was considered positive in tumour cells of 242 of 451 (53.7%) GCs. One hundred fourteen of these 242 (47.1%) GCs also showed CLDN18.2 expression, demonstrating a significant positive correlation (*p* = 0.007). We also tested αvβ3 and, interestingly, no correlation was found (*p* = 0.588). Of 446 GCs, 131 (29.3%) were considered positive for expression of the epithelial cellular adhesion molecule (EpCAM) extracellular domain (EpEX). Of these 131 EpEX-positive GCs, a highly significantly increased number was CLDN18.2-negative (93 of 131, 71%), suggesting an inverse correlation. The intracellular domain of EpCAM (EpICD), as well as the adherens junction protein E-cadherin, showed no correlation with CLDN18.2.

### CLDN18.2 is frequently heterogeneously expressed in GC

In our cohort, nearly all tumours with strong immunostaining also comprised negative tumour cells, demonstrating an expression phenotype of CLDN18.2 with high tendencies towards heterogeneity. To emphasize the extent of heterogeneity, we considered those tumours to show a strong heterogeneity phenotype that displayed both strongly stained and negative tumour cells in at least half of the tumour tissue combined. Thirty-two tumours (6.7% of 481) met those criteria.

These tumours were closely investigated for different distribution patterns of heterogeneity. Five of 32 (15.6%) GCs showed a “downward gradient” with weaker staining intensity towards the depth of the tumour. Sixteen of 32 (50%) tumours showed a patchy distribution of different immunostaining intensities. The remaining cases (11 of 32; 34.4%) displayed a scattered pattern, with randomly distributed cells of different staining intensities. Representative images are displayed in Online Resource 1.

### CLDN18.2 and survival

**Fig. 3:**
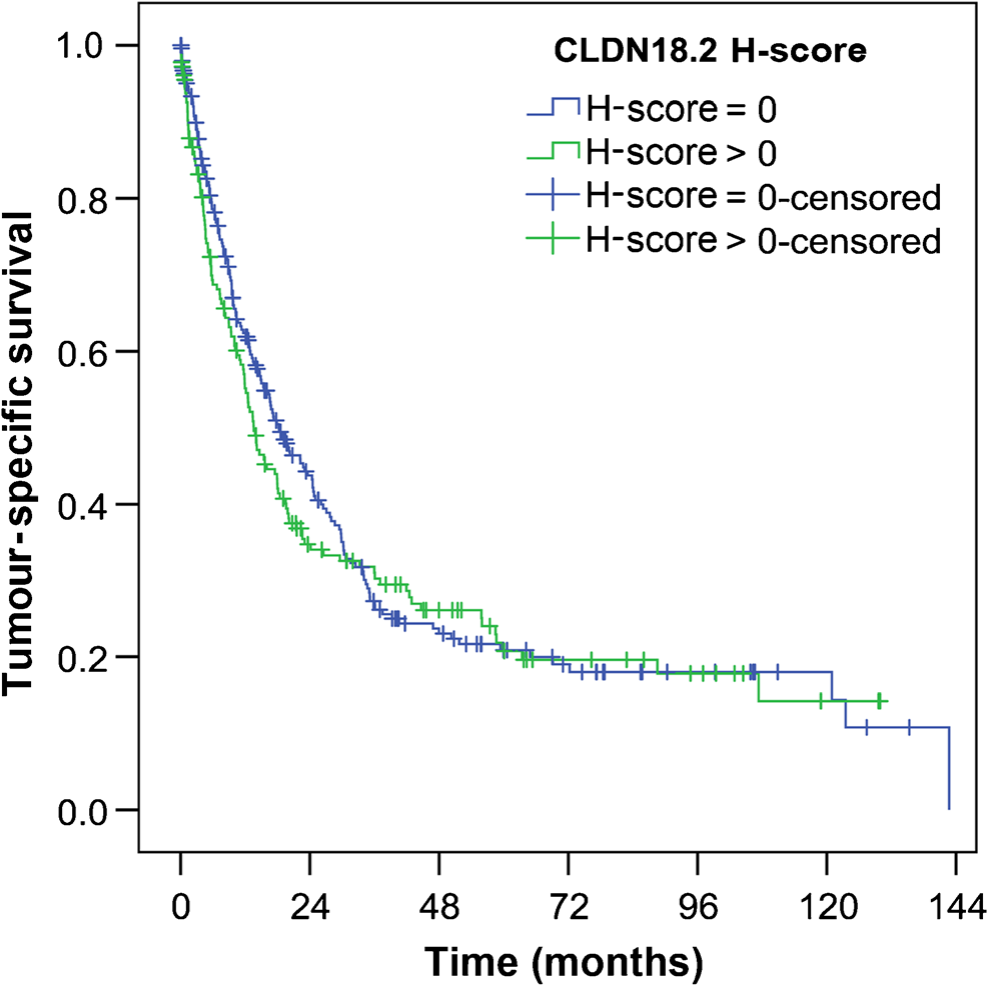
CLDN18.2 and survival. There was no significant correlation between tumour-specific survival and CLDN18.2 expression in tumour cells (249 vs. 181 patients; median survival 18.2 vs. 13.6 months; *p* = 0.439). *p* value was obtained via log-rank test

Tumour-specific survival data was available in a total of 430 patients. We found no correlation between CLDN18.2 expression and tumour-specific survival (Fig. 3). However, we saw haphazard tendencies of R1/2-resected carcinomas to express CLDN18.2 more frequently, which had lost significance after appliance of the multiple testing procedure as well as multivariate analysis (Table 1). Hence, we reviewed the survival curves of patients with solely R0-resected carcinomas (Online Resource 2A). Additionally, we tested for the impact of different CLDN18.2 expression intensities and different cancer stages (Online Resource 2B-F). We found no significant correlation of CLDN18.2 with survival in any of these cases.

## Discussion

### EBV infection is associated with strong CLDN18.2 expression

EBV-associated GC (EBVaGC) is a unique etiological entity, characterized by distinct features such as male predominance, predisposition to the proximal stomach, and relative favourable prognosis [17]. Several lines of evidence indicate that EBV infection into epithelial cells is mediated by cell-to-cell contact, while it has been suggested that the presence of extensive cell junctions may restrict antibody accessibility [18, 19]. Our data not only show that CLDN18.2 expression is correlated with EBVaGC, as it was previously implied by other studies [20], but also that the magnitude of its expression appears to play an important role for EBV in GC, shown by the fact that most EBVaGCs expressed CLDN18.2 at astoundingly high levels. Preserved expression of CLDN18.2 in tumour cells is likely linked to key features of EBV-mediated carcinogenesis. Recently described heterogeneous EBV distribution in GC [9] may also suggest roles of yet unknown factors ensuring EBV maintenance in tumour cells.

### Mucin phenotype correlates with CLDN18.2 expression

Expression of various mucins has been used to classify GC into different mucin phenotypes. We found CLDN18.2 to significantly correlate with mucin phenotypes, the intestinal phenotype predominantly showing downregulation of CLDN18.2. Similar findings have been previously described, suggesting the loss of CLDN18 to be involved in the pathogenesis of the intestinal phenotype and indicating its expression as a marker for the gastric phenotype [21, 22].

Of note, we found no correlation between CLDN18.2 expression and the Laurén classification. This is consistent with results obtained by Jun et al. [23], and in contrast with Sahin et al. [4], who found significantly less CLDN18.2 expression in intestinal compared with diffuse-type GC. However, in our study, CLDN18.2 did correlate with LYZ expression, which has recently been shown to be linked to diffuse-type GC [6]. Due to a substantial overlap of expression, our data underpins that CLDN18.2 may not be decisively attributable to a specific GC subtype, but could rather be differently involved in the pathogenesis and invasion progression of distinct GC subtypes.

### CLDN18.2 expression shows no correlation with survival

We did not find a correlation between CLDN18.2 expression and survival. In contrast to our results, two previous studies have suggested that reduced CLDN18.2 expression correlates with poor prognosis. This was reported from a 134 patient cohort [23] and a 65 patient cohort with advanced GCs [21]. Hence, CLDN18.2 was presumed to resemble an independent prognostic marker. The data we obtained from our cohort of 481 patients does not support these previous indications. In contrast, patients with tumours expressing CLDN18.2 at different levels showed no congruent survival phenotype tendencies, whatsoever (Online Resource 2B).

### CLDN18.2 is linked to other cell junction proteins

Previous studies have depicted the critical role of cell junction dysregulation in malignant transformation and invasion [24]. Interaction of different cell junction proteins has for example been demonstrated in human lung cancer cells, where claudin-7 and integrin β1 form a complex regulating cell growth and cell cycle progression [25]. We demonstrate a correlation of CLDN18.2 and the integrin αvβ5 in GC, which may provide ground for further studies to investigate the connection between these two potential antibody targets.

The epithelial cellular adhesion molecule (EpCAM) is composed of an extracellular (EpEX) and an intracellular domain (EpICD), which are separated upon cleavage. EpICD is released into the cytoplasm to trigger oncogenic signalling, while EpEX is shedded [26]. In human colon cancer cells, EpCAM has been suggested to regulate tight junctions by degradation of selected claudins [27]. Hence, the inverse correlation of CLDN18.2 and EpEX we describe in GC is particularly interesting. However, since we found no correlation with EpICD, EpEX may also have distinct functions, which link it to CLDN18.2. Recently, EpEX has been shown to serve as a soluble agonist to promote cell migration and proliferation through activation of the EGFR pathway in colon cancer [28]. Intriguingly, a direct interaction of CLDN18 with the EGFR pathway has also recently been proposed in bile duct neoplasia [29]. Hence, EGFR signalling may also play a role for CLDN18.2 expression in GC and should therefore be of interest to future studies.

### CLDN18.2 heterogeneity poses a challenge to diagnostic evaluations

Previous studies have addressed diagnostic insecurities that may arise from heterogeneous CLDN18.2 immunostaining [30]. On the basis of distributions of H-scores and IHC-scores in our cohort, we also demonstrate a high prevalence of CLDN18.2 heterogeneity in our study (Fig. 2a). Furthermore, we depict heterogeneity patterns, which may amount to great challenges in clinical practice and scientific research (Online Resource 1). While a scattered distribution of staining intensities may still enable a proper and representative assessment of the tumour in a small tissue specimen, a patchy pattern may lead the examiner towards a severe misjudgement of the overall expression rate. Additionally, we describe a frequent occurrence of declining immunostaining intensity towards the invasive front and therefore within the depth of the tumour tissue. This may be exceptionally problematic for biopsy examinations, which predominantly allow assessment of shallow parts of the tumour. Of note, this observed heterogeneity pattern may be linked to previous findings, suggesting an inverse correlation between CLDN18.2 expression and the invasive potential [16].

### CLDN18.2 expression is decreased in gastric cancer

The majority of GCs of our cohort was completely devoid of any CLDN18.2 expression, while a large proportion of positive GCs showed solely weak staining levels. The specificity of our antibody was frequently demonstrated by a strong staining of the normal gastric mucosa and loss of staining in intestinal metaplasia.

A low expression level of claudin proteins in tumour cells is compliant with the concept of destruction of tight junctions leading to disruption of epithelial cell cohesion and promoting cell invasiveness [31]. Congruent with this and supporting to our findings, various previous studies have also implicated CLDN18 downregulation as a characteristic of GC [16, 21, 23, 32, 33].

However, Sahin et al. [4] reported CLDN18.2 expression in 77% of GCs (51/66 patients) and significant labelling (defined as ≥ 60% of tumour cells displaying ≥ 2+ staining intensity) in 56% of GCs (37/66 patients). In the previously performed FAST study [5], significant CLDN18.2 expression (defined as ≥ 40% of tumour cells displaying ≥ 2+ staining intensity) was shown in 48% (334/686 patients). Recently published results from a Japanese study cohort even displayed 52% significant expression (135/262 patients) using the same criteria [34]. In comparison, when applying the criteria of the FAST study to our data, we obtain 10% GCs with significant expression (48/481, see Online Resource 3 for clinico-pathological characteristics). The remarkable divergence of CLDN18 expression rates across studies may be related to ethnic characteristics or linked to intratumoural GC heterogeneity, especially with respect to limitations of small tissue specimens. Furthermore, well-defined immunostaining and scoring approaches are crucial for generating comparable data.

In conclusion, our study provides a detailed illustration and description of CLDN18.2 expression and its correlation with various clinico-pathological factors in GC, using appropriate tissue specimens and a Caucasian cohort of considerable size. We conclude that further studies will be needed in order to establish CLDN18.2 in future GC therapy.

## Electronic supplementary material

Online Resource 1 CLDN18.2 heterogeneity patterns Representative images of heterogeneity patterns in tumours with intestinal type (Laurén) are shown. (A) Downward gradient: declining immunostaining intensity towards the invasive front. (B) Patchy pattern: Circumscribed adjacent areas with strong and weak immunostaining. (C) Scattered pattern: Randomly distributed cells with different immunostaining intensities. Original magnification of the left column is 50-fold. Original magnification of rounded images is 400-fold. (JPG 2852 kb)

Online resource 2 Kaplan-Meier curves stratified according to R status, expression intensity and cancer stages (A) There was no significant correlation between tumour-specific survival and CLDN18.2 expression in patients with R0-resected tumours (221 vs. 144 patients; median survival 22.2 vs. 17.9 mo; *p* = 0.751) (B) The cohort was split into three groups showing negative, low or high expression of CLDN18.2. High expression was defined as ≥40% of tumour cells showing ≥2+ (criteria for significant expression of the FAST study). There was no significant correlation between tumour-specific survival and different CLDN18.2 expression intensities (249 vs 137 vs 44 patients, median survival 18.2 vs. 14.6 vs. 10.5 mo, *p* = 0.070). (C – F) Patients were stratified according to UICC stage. No significant survival correlation was found in any cancer stage of patients with tumours showing no or any CLDN18.2 expression. (C) 40 vs. 28 patients in stage I disease with median survival 102.9 vs. (−) mo (*p* = 0.710). (D) 56 vs. 39 patients in stage II disease with median survival 30.2 vs. 55.9 mo (*p* = 0.713). (E) 109 vs. 71 patients in stage III disease with median survival 12.7 vs. 11.6 mo (*p* = 0.847). (F) 42 vs. 42 patients in stage IV disease with median survival 8.0 vs. 5.7 mo (*p* = 0.111). *p*-values were obtained via log-rank-test. (JPG 1416 kb)

Online resource 3 (DOCX 40 kb)
