# Supplementary figures and images for: Expression of the potential therapeutic target claudin-18.2 is frequently decreased in gastric cancer: results from a large Caucasian cohort study
Source: Virchows Arch. 2019 Jul 22;475(5):563–71. doi: 10.1007/s00428-019-02624-7 (PMC6861347; doi:10.1007/s00428-019-02624-7)

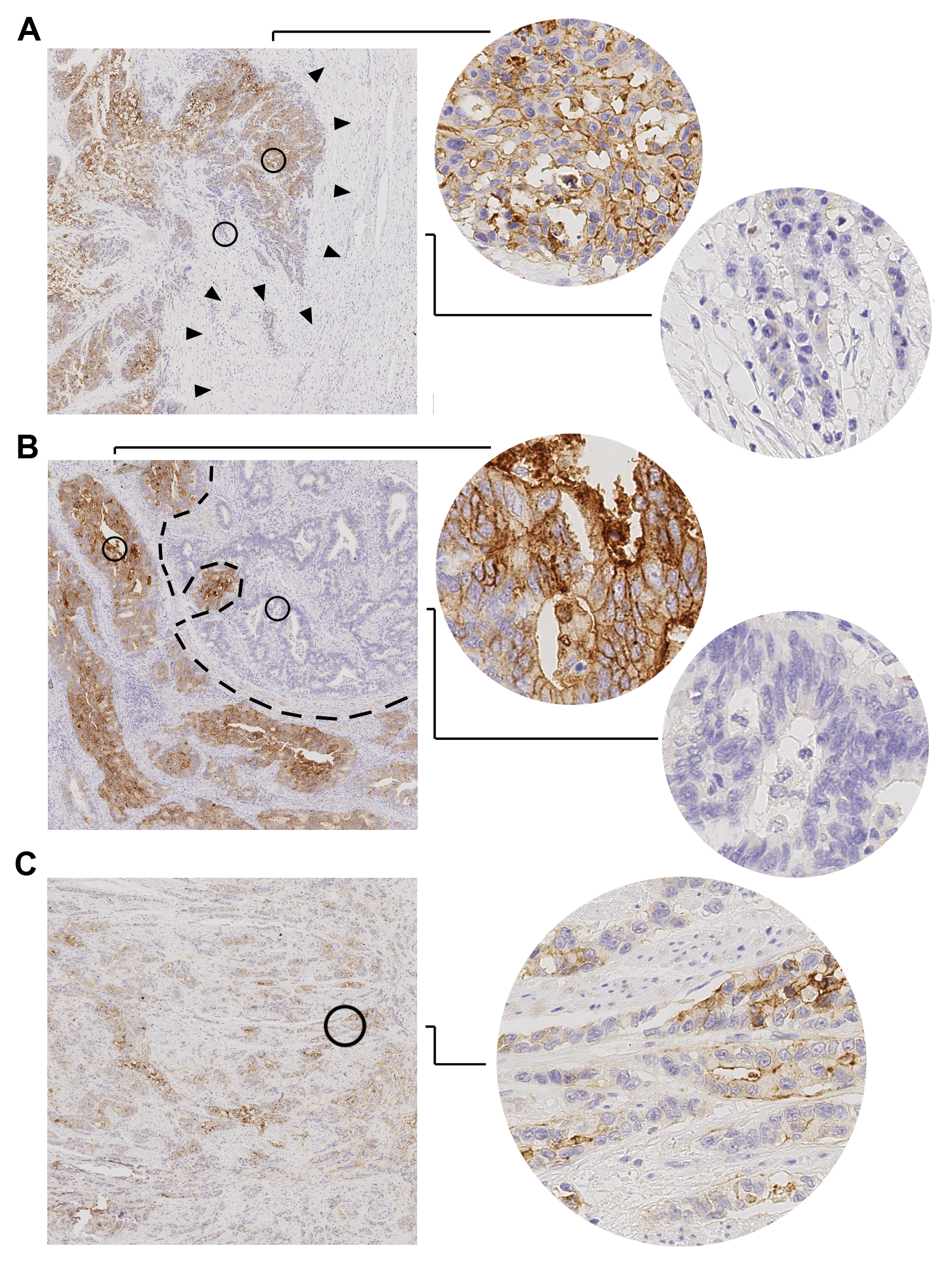

Supplement: Supplementary file 1 — CLDN18.2 heterogeneity patterns Representative images of heterogeneity patterns in tumours with intestinal type (Laurén) are shown. (A) Downward gradient: declining immunostaining intensity towards the invasive front. (B) Patchy pattern: Circumscribed adjacent areas with strong and weak immunostaining. (C) Scattered pattern: Randomly distributed cells with different immunostaining intensities. Original magnification of the left column is 50-fold. Original magnification of rounded images is 400-fold. (JPG 2852 kb) [file 428_2019_2624_MOESM1_ESM.jpg]

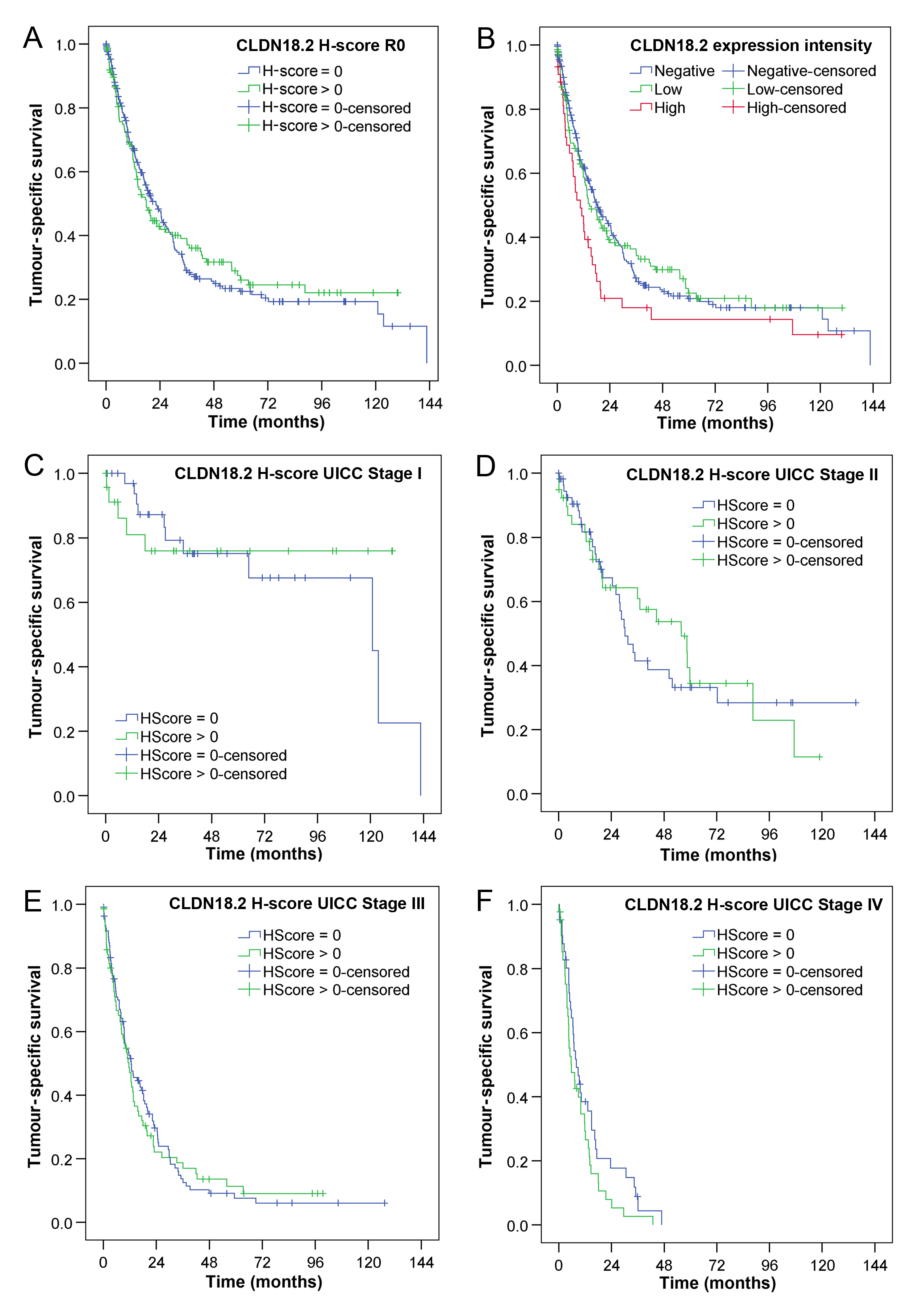

Supplement: Supplementary file 2 — Kaplan-Meier curves stratified according to R status, expression intensity and cancer stages (A) There was no significant correlation between tumour-specific survival and CLDN18.2 expression in patients with R0-resected tumours (221 vs. 144 patients; median survival 22.2 vs. 17.9 mo; p = 0.751) (B) The cohort was split into three groups showing negative, low or high expression of CLDN18.2. High expression was defined as ≥40% of tumour cells showing ≥2+ (criteria for significant expression of the FAST study). There was no significant correlation between tumour-specific survival and different CLDN18.2 expression intensities (249 vs 137 vs 44 patients, median survival 18.2 vs. 14.6 vs. 10.5 mo, p = 0.070). (C – F) Patients were stratified according to UICC stage. No significant survival correlation was found in any cancer stage of patients with tumours showing no or any CLDN18.2 expression. (C) 40 vs. 28 patients in stage I disease with median survival 102.9 vs. (−) mo (p = 0.710). (D) 56 vs. 39 patients in stage II disease with median survival 30.2 vs. 55.9 mo (p = 0.713). (E) 109 vs. 71 patients in stage III disease with median survival 12.7 vs. 11.6 mo (p = 0.847). (F) 42 vs. 42 patients in stage IV disease with median survival 8.0 vs. 5.7 mo (p = 0.111). p-values were obtained via log-rank-test. (JPG 1416 kb) [file 428_2019_2624_MOESM2_ESM.jpg]
