# Supplementary material for: Expression of the potential therapeutic target claudin-18.2 is frequently decreased in gastric cancer: results from a large Caucasian cohort study
Source: Virchows Arch. 2019 Jul 22;475(5):563–71. doi: 10.1007/s00428-019-02624-7 (PMC6861347; doi:10.1007/s00428-019-02624-7)
Supplement: Supplementary file 3 — (DOCX 40 kb) [file 428_2019_2624_MOESM3_ESM.docx]

**Supplementary Table 1**:

CLDN18.2 expression intensity and correlation with clinico-pathological patient characteristics

| **Characteristic** | **Variable** | **Total valid**  **[N (%)]** | | **CLDN18.2 expression** | | | | | | **p-value** |  |
| --- | --- | --- | --- | --- | --- | --- | --- | --- | --- | --- | --- |
|  |  |  |  | **High** | | **Low** | | **Negative** | |  |  |
|  |  |  | | **[N (%)]** | | **[N (%)]** | | **[N (%)]** | |  |  |
| Age | <68 | 234 | (49.4) | 29 | (12.4) | 79 | (33.8) | 126 | (53.8) | 0.053 |  |
|  | ≥68 | 240 | (50.6) | 19 | (7.9) | 73 | (30.4) | 148 | (61.7) |  |  |
| Gender | Female | 179 | (37.2) | 12 | (6.7) | 60 | (33.5) | 107 | (59.8) | 0.292 |  |
|  | Male | 302 | (62.8) | 36 | (11.9) | 95 | (31.3) | 171 | (56.6) |  |  |
| Localization | Proximal | 145 | (31.0) | 17 | (11.7) | 43 | (29.7) | 85 | (58.6) | 0.969 |  |
|  | Distal | 323 | (69.0) | 29 | (9.0) | 108 | (33.4) | 186 | (57.6) |  |  |
| Laurén phenotype | Intestinal | 252 | (52.5) | 30 | (11.9) | 84 | (33.3) | 138 | (54.8) | 0.359 |  |
|  | Diffuse | 145 | (30.2) | 12 | (8.3) | 43 | (29.7) | 90 | (62.1) |  |  |
|  | Mixed | 31 | (6.5) | 2 | (6.5) | 14 | (45.2) | 15 | (48.4) |  |  |
|  | Unclassified | 52 | (10.8) | 4 | (7.7) | 13 | (25.0) | 35 | (67.3) |  |  |
| Mucin type | Intestinal | 122 | (29.0) | 6 | (4.9) | 23 | (18.9) | 93 | (76.2) | < 0.001 | * |
|  | Gastric | 64 | (15.2) | 7 | (10.9) | 25 | (39.1) | 32 | (50.0) |  |  |
|  | Mixed | 162 | (38.5) | 20 | (12.3) | 58 | (35.8) | 84 | (51.9) |  |  |
|  | Unclassified | 73 | (17.3) | 13 | (17.8) | 28 | (38.4) | 32 | (43.8) |  |  |
| T-category | T1 | 58 | (12.1) | 3 | (5.2) | 22 | (37.9) | 33 | (56.9) | 0.588 |  |
|  | T2 | 53 | (11.0) | 6 | (11.3) | 15 | (28.3) | 32 | (60.4) |  |  |
|  | T3 | 195 | (40.6) | 20 | (10.3) | 61 | (31.3) | 114 | (58.5) |  |  |
|  | T4 | 174 | (36.3) | 19 | (10.9) | 57 | (32.8) | 98 | (56.3) |  |  |
| N-category | N0 | 136 | (28.5) | 11 | (8.1) | 49 | (36.0) | 76 | (55.9) | 0.533 |  |
|  | N1 | 67 | (14.0) | 8 | (11.9) | 19 | (28.4) | 40 | (59.7) |  |  |
|  | N2 | 84 | (17.6) | 4 | (4.8) | 25 | (29.8) | 55 | (65.5) |  |  |
|  | N3 | 190 | (39.8) | 25 | (13.2) | 61 | (32.1) | 104 | (54.7) |  |  |
| M-category | 0 | 387 | (80.5) | 37 | (9.6) | 122 | (31.5) | 228 | (58.9) | 0.300 |  |
|  | 1 | 94 | (19.5) | 11 | (11.7) | 33 | (35.1) | 50 | (53.2) |  |  |
| UICC-stage | I | 78 | (16.4) | 7 | (9.0) | 26 | (33.3) | 45 | (57.7) | 0.632 |  |
|  | II | 107 | (22.4) | 10 | (9.3) | 36 | (33.6) | 61 | (57.0) |  |  |
|  | III | 198 | (41.5) | 20 | (10.1) | 59 | (29.8) | 119 | (60.1) |  |  |
|  | IV | 94 | (19.7) | 11 | (11.7) | 33 | (35.1) | 50 | (53.2) |  |  |
| L-category | L0 | 214 | (48.1) | 21 | (9.8) | 67 | (31.3) | 126 | (58.9) | 0.729 |  |
|  | L1 | 231 | (51.9) | 23 | (10.0) | 76 | (32.9) | 132 | (57.1) |  |  |
| V-category | V0 | 394 | (88.7) | 39 | (9.9) | 121 | (30.7) | 234 | (59.4) | 0.349 |  |
|  | V1 | 50 | (11.3) | 3 | (6.0) | 22 | (44.0) | 25 | (50.0) |  |  |
| Grading | G1/G2 | 116 | (24.3) | 9 | (7.8) | 40 | (34.5) | 67 | (57.8) | 0.810 |  |
|  | G3/G4 | 361 | (75.7) | 39 | (10.8) | 114 | (31.6) | 208 | (57.6) |  |  |
| R-status | R0 | 402 | (87.2) | 35 | (8.7) | 125 | (31.1) | 242 | (60.2) | 0.007 | * |
|  | R1 & 2 | 59 | (12.8) | 11 | (18.6) | 22 | (37.3) | 26 | (44.1) |  |  |
| H. pylori-status | Negative | 341 | (84.8) | 36 | (10.6) | 107 | (31.4) | 198 | (58.1) | 0.980 |  |
|  | Positive | 61 | (15.2) | 5 | (8.2) | 21 | (34.4) | 35 | (57.4) |  |  |
| EBV-status | Negative | 445 | (95.5) | 38 | (8.5) | 141 | (31.7) | 266 | (59.8) | <0.001 | * |
|  | Positive | 21 | (4.5) | 10 | (47.6) | 7 | (33.3) | 4 | (19.0) |  |  |
| MSI-status | MSS | 429 | (92.3) | 45 | (10.5) | 134 | (31.2) | 250 | (58.3) | 0.934 |  |
|  | MSI | 36 | (7.7) | 3 | (8.3) | 12 | (33.3) | 21 | (58.3) |  |  |
| HER2-status | Negative | 400 | (92.2) | 44 | (11.0) | 129 | (32.3) | 227 | (56.8) | 0.022 | ^1^ |
|  | Positive | 34 | (7.8) | 1 | (2.9) | 7 | (20.6) | 26 | (76.5) |  |  |
| MET-status | Negative | 434 | (92.9) | 43 | (9.9) | 137 | (31.6) | 254 | (58.5) | 0.290 |  |
|  | Positive | 33 | (7.1) | 4 | (12.1) | 13 | (39.4) | 16 | (48.5) |  |  |
| EpEX | Negative | 315 | (70.6) | 42 | (13.3) | 107 | (34.0) | 166 | (52.7) | < 0.001 | * |
|  | Positive | 131 | (29.4) | 5 | (3.8) | 33 | (25.2) | 93 | (71.0) |  |  |
| EpICD | Negative | 142 | (31.6) | 16 | (11.3) | 48 | (33.8) | 78 | (54.9) | 0.442 |  |
|  | Positive | 307 | (68.4) | 32 | (10.4) | 94 | (30.6) | 181 | (59.0) |  |  |
| E-Cadherin | Negative | 324 | (73.1) | 33 | (10.2) | 99 | (30.6) | 192 | (59.3) | 0.382 |  |
|  | Positive | 119 | (26.9) | 14 | (11.8) | 40 | (33.6) | 65 | (54.6) |  |  |
| αvβ3 integrin | Negative | 336 | (74.2) | 35 | (10.4) | 107 | (31.8) | 194 | (57.7) | 0.625 |  |
|  | Positive | 117 | (25.8) | 12 | (10.3) | 34 | (29.1) | 71 | (60.7) |  |  |
| αvβ5 integrin | Negative | 209 | (46.3) | 18 | (8.6) | 54 | (25.8) | 137 | (65.6) | 0.008 |  |
|  | Positive | 242 | (53.7) | 29 | (12.0) | 85 | (35.1) | 128 | (52.9) |  |  |
| Lysozyme | Negative | 217 | (50.5) | 14 | (6.5) | 52 | (24.0) | 151 | (69.6) | < 0.001 | * |
|  | Positive | 213 | (49.5) | 32 | (15.0) | 83 | (39.0) | 98 | (46.0) |  |  |

High expression is defined as ≥ 40% of tumour cells showing ≥ 2+ staining intensity (criteria of the FAST study for significant expression). Low expression is attributed to all other tumours with ≥ 10% of tumour cells showing any expression. p-values obtained via Kendall’s tau or Fisher’s exact test.; *Significant after multiple testing correction; ^1^ Loss of significance after multiple testing correction.
